# Thermodynamic Analysis of a Rankine Cycle Powered Vapor Compression Ice Maker Using Solar Energy

**DOI:** 10.1155/2014/742606

**Published:** 2014-08-17

**Authors:** Bing Hu, Xianbiao Bu, Weibin Ma

**Affiliations:** ^1^Guangzhou Institute of Energy Conversion, Key Laboratory of Renewable Energy, Chinese Academy of Sciences, Guangzhou 510640, China; ^2^University of Chinese Academy of Sciences, Beijing 100049, China

## Abstract

To develop the organic Rankine-vapor compression ice maker driven by solar energy, a thermodynamic model was developed and the effects of generation temperature, condensation temperature, and working fluid types on the system performance were analyzed. The results show that the cooling power per square meter collector and ice production per square meter collector per day depend largely on generation temperature and condensation temperature and they increase firstly and then decrease with increasing generation temperature. For every working fluid there is an optimal generation temperature at which organic Rankine efficiency achieves the maximum value. The cooling power per square meter collector and ice production per square meter collector per day are, respectively, 126.44 W m^−2^ and 7.61 kg m^−2^ day^−1^ at the generation temperature of 140°C for working fluid of R245fa, which demonstrates the feasibility of organic Rankine cycle powered vapor compression ice maker.

## 1. Introduction

In recent years, there is an increasing need for cooling due to global warming, so, the energy consumption used for cooling has increased drastically [1, 2]. The use of solar energy is one important contribution for the reduction of fossil fuel consumption and harmful emissions to the environment, while solar cooling for food, beverage, and seafood preservation or air-conditioning is an attractive application of solar energy because both the insolation supply and the need for refrigeration reach maximum levels in the same period. In particular in some places, such as Tibet in China, a large proportion of people live in rural or remote locations where electricity is presently far from sufficient; also the solar radiation is the most sufficient in those areas and refrigeration device driven by solar energy is a very useful application for food and vaccine preservation. Solar powered ice makers or refrigerators have been reported by a lot of researchers [3–5]. Among these researches, there are many different ways to convert solar energy into cooling processes [6–9]; these are by the use of the absorption/adsorption refrigeration cycle and the organic Rankine cycle/vapor compression cycle (ORC/VCC) [10–12]. Boubakri [13, 14] carried out tests on an adsorptive solar powered ice maker using methanol/carbon pair. Vasta et al. [15] presented a model for dynamic simulation of an adsorptive ice maker and showed that the ice maker is able to freeze 5 kg of water during all days of June. Wang et al. [16] described the working principle of the combined cycles of solar refrigeration and heating, and their experimental results showed that the hybrid system is capable of heating 60 kg of water to about 90°C as well as producing ice at 10 kg per day with a 2 m^2^ solar collector. Li et al. [17] developed a no valve, flat plate solar ice maker and carried out experimental tests under both indoor and outdoor. Sumathy and Zhongfu [18] presented a solar-powered ice maker with the solid adsorption pair of activated carbon and methanol. In this paper, a simple flat plate collector with an exposed area of 0.92 m^2^ was employed to produce ice of about 4-5 kg day^−1^. Li and Wang [19] presented a uniform pressure model to describe the heat and mass transfer in an adsorbent bed for a flat plate solar ice maker. Kiplagat et al. [20] proposed consolidated composite material made from expanded graphite powder impregnated with LiCl salt for use in solar powered adsorption ice makers. Freni et al. [21] presented the simulation results of an adsorptive ice maker system driven by solar energy. Leite and Daguenet [22] studied the performance of an adsorption solar cooling system using the activated carbon-methanol pair and its numerical simulation. Rivera et al. [23] developed a novel solar intermittent refrigeration system for ice production with ammonia/lithium nitrate mixture as absorption working pairs. Today, most solar refrigeration devices on the market use absorption or adsorption refrigeration to achieve the cooling effect; however, these devices are generally costly and huge.

Currently, the use of thermal energy to operate an ORC/VCC for air-conditioning and cooling has become the subject of renewed interest and has been reported by several investigators [24]. Aphornratana and Sriveerakul [25] theoretically analyzed a combined Rankine vapor compression refrigeration cycle powered by low grade thermal energy. Wang et al. [26, 27] introduced a novel thermally activated cooling concept; a combined cycle couples an ORC and a VCC and developed a prototype with nominal cooling capacity of 5 kW. Demierre et al. [28] presented the development of an ORC-ORC prototype with HFC-134a as working fluid and heating power about 20 kW at the condenser. ORC/VCC solar cooling systems convert collected solar heat into a cooling effect, which is accomplished at the site of the installation by using the Rankine cycle to generate the shaft work required to drive a vapor compression cycle. However, few people studied ice making using ORC/VCC, especially ice making utilizing solar energy. The biggest difference between ice making and air conditioning is that the evaporation temperature for ice making is about −5°C, which is lower than that for air-conditioning, leading to low efficiency and high pressure ratio for compressor used in ice maker. ORC/VCC provides an alternative to solar ice making besides absorption and adsorption ice making; so, a feasibility demonstration of making use of ORC/VCC driven by solar energy for ice making is of great significance.

In a typical ORC/VCC, the maximum temperature at the inlet to the expander is limited to the temperature of the fluid leaving the solar collector. Because of the relatively low temperature capability associated with the flat plate collectors the coefficient of performance (COP) of an ORC/VCC is generally low. In this paper, to improve the COP of an ORC/VCC for ice making driven by solar energy, the parabolic trough collectors with higher temperature are selected to provide energy to heat and vaporize a working fluid, and a stationary modeling is developed in order to demonstrate the feasibility of such a cooling process.

## 2. System Design

**Figure 1:**
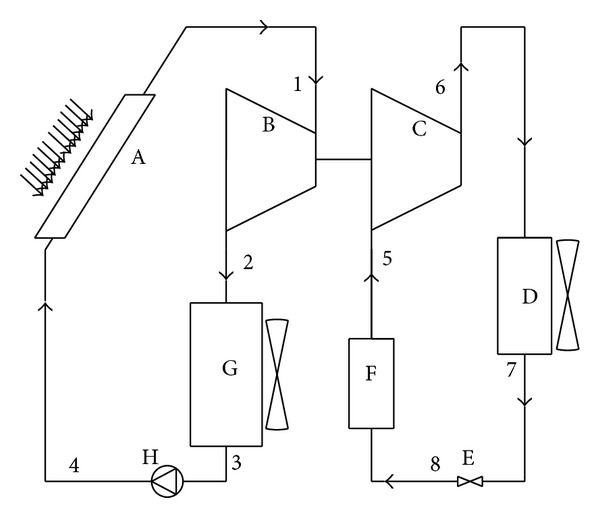
The system schematic drawing. A: solar collector (generator), B: expander, C: compressor, D: condenser for cooling system, E: throttle valve, F: evaporator for ice maker, G: condenser for power system, and H: working fluid pump.

The system of ORC/VCC for ice making driven by solar energy mainly consists of solar collector (generator), expander, compressor, condenser for cooling system, throttle valve, evaporator for ice maker, condenser for power system, and working fluid pump, as shown in Figure 1. The working principle of this system is as follows: a solar collector is used to provide energy to heat and vaporize a working fluid with low boiling point. Energy is extracted from this vapor in an expansion engine that is used to drive a vapor compressor. The fluid exiting the expander is condensed and pumped back to the boiler pressure where it is again vaporized, as shown in Figure 1. R600, R245fa, and R600A are separately selected as the working fluids to compare their cycle efficiency and to select the suitable working fluids for the solar ice maker. The same working fluid is used for both ORC and VCC to avoid the gas separation caused by leakage. In Figure 1, ORC is also called power system and VCC is also an ice making system. The parabolic trough collectors are selected due to having high efficiency at much higher collecting temperature and solar collector is also used as generator. Considering the instability of solar heat source, the radial and axial flow expander is employed, which can suit the variability of heat source. To improve the drive efficiency, a direct drive without gear and coupling is used between expander and compressor.

## 3. Thermodynamic Analysis

To develop the thermodynamic model, the following assumptions are made:

1. friction and heat losses in ORC/VCC are negligible;
2. the power consumed by condensers for both ORC and VCC is negligible.

For ORC,

(1) $$\begin{array}{c} W_{\exp}=m_{p}\left(h_{1}-h_{2s}\right)\eta_{\exp},\\ W_{\text{pump}}=m_{p}\frac{\left(h_{4s}-h_{3}\right)}{\eta_{\text{pump}}},\\ Q_{\text{boi}}=m_{p}\left(h_{1}-h_{4}\right),\\ W_{\text{net}}=W_{\exp}-W_{\text{pump}},\\ \eta_{p}=\frac{W_{\text{net}}}{Q_{\text{boi}}}. \end{array}$$

For VCC,

(2) $$\begin{array}{c} Q_{\text{eva}}=m_{c}\left(h_{5}-h_{8}\right),\\ W_{\text{com}}=m_{c}\frac{\left(h_{6s}-h_{5}\right)}{\eta_{\text{com}}},\\ W_{\text{com}}=W_{\exp},\\ \text{CO}{\text{P}}_{c}=\frac{Q_{\text{eva}}}{W_{\text{com}}+W_{\text{pump}}}. \end{array}$$

The overall COP of RC/VCC is defined as

(3) $$\begin{array}{c} \text{CO}{\text{P}}_{s}=\eta_{p}\text{CO}{\text{P}}_{c}, \end{array}$$

(4) $$\begin{array}{c} P_{c}=\frac{Q_{\text{eva}}}{n}, \end{array}$$

(5) $$\begin{array}{c} N=\frac{Q_{\text{eva}}}{nh_{\text{ice}}}\times 3600\times 7, \end{array}$$

(6) $$\begin{array}{c} \eta_{t}=\eta_{\text{solar}}\text{CO}{\text{P}}_{s}, \end{array}$$

(7) $$\begin{array}{c} \eta_{\text{solar}}=0.762-0.2125\left(\frac{\Delta T}{G_{B}}\right)-0.001672\left(\frac{\Delta T^{2}}{G_{B}}\right), \end{array}$$

(8) $$\begin{array}{c} \Delta T=T_{\text{boi}}-T_{c}. \end{array}$$

## 4. Discussions

Considering the instability of solar heat source, the generation temperature *T*_boi_ is in the range of 60–160°C, the condensation temperature is 35–45°C, and the evaporation temperature is −5°C and keeps being invariable. For evaluating the feasibility of ORC/VCC for ice maker, the intensity of solar direct radiation is 300 W m^−2^ and the total solar direct radiation is 7560 kJ m^−2^ per day. The isentropic efficiencies for expander, compressor, and working fluid pump are, respectively, 0.85, 0.8, and 0.9, as shown in Table 1.

### 4.1. Effect of Generation Temperature on System Performance

**Figure 2:**
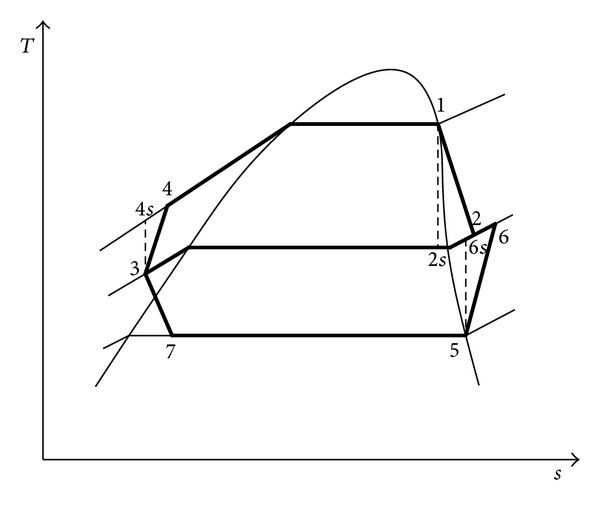
*T*-*s* diagram of the ORC/VCR cycle.

**Figure 3:**
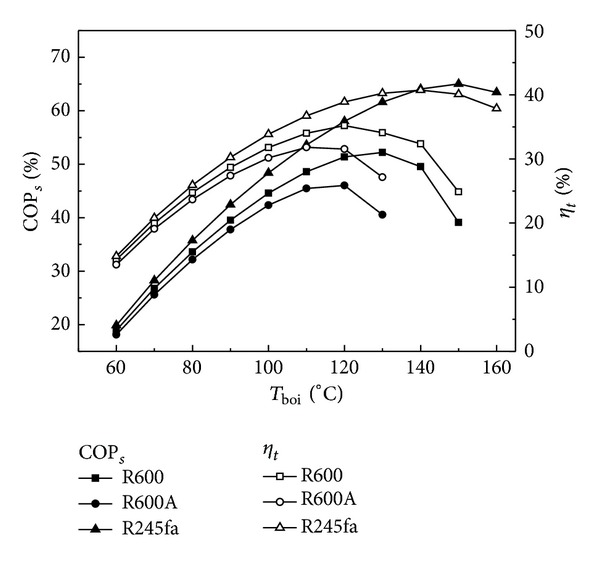
Effects of *T*_boi_ on COP_*s*_ and *η*_*t*_.

Figure 2 shows COP_*s*_ and *η*_*t*_ as a function of *T*_boi_. In Figure 2, the condensation temperature and evaporation temperature are, respectively, 40 and −5°C. COP_*s*_ and *η*_*t*_ depend largely on *T*_boi_ and they increase firstly and then decrease with increasing *T*_boi_, as shown in Figure 2. The effects of *T*_boi_ on *P*_*c*_ and *N* are illustrated in Figure 3. Observing the profiles from Figures 2 and 3 it is obvious that COP_*s*_, *η*_*t*_, *P*_*c*_, and *N* have the same change trend with *T*_boi_. For solar collector, the thermal efficiency, *η*_solar_, depends on both the solar radiation and the temperature difference between the generation temperature and ambient. When solar radiation, condensation temperature, and ambient temperature keep being invariable, *η*_solar_ only depends on the generation temperature which can be controlled by changing the mass flow rate of working fluid for ORC. That is to say, *η*_solar_ depends on the mass flow rate of working fluid for ORC. According to (7) and (8), the higher the *T*_boi_, the lower the *η*_solar_. The coefficient of performance for compressor, COP_*c*_, keeps being invariable when the condensation temperature and evaporation temperature remain unchanged. Therefore, a conclusion can be drawn through the above analysis that there is always an optimal *T*_boi_ at which *P*_*c*_ and *N* can achieve the maximum values when solar radiation, condensation temperature, and evaporation temperature keep being invariable, as shown in Figure 3. The optimal *T*_boi_ is different for different working fluids, and it is 120, 140, and 120°C for working fluids of R600, R245fa, and R600A, respectively (Figure 7). *P*_*c*_ is, respectively, 110.22, 126.44, and 103.13 W m^−2^ and *N* is, respectively, 6.64, 7.61, and 6.21 kg m^−2^ day^−1^ at the optimal *T*_boi_ for working fluids of R600, R245fa, and R600A, indicating that the working fluid R245fa has the optimal ice making performance compared with two other working fluids.

The programmable logic controller and frequency converter are suggested to be adopted in practical use and the optimal *T*_boi_ can automatically be calculated by collecting solar radiation, condensation temperature, and evaporation temperature and then the mass flow rate of working fluid for ORC can automatically be adjusted by the frequency converter, working fluid pump, and programmable logic controller so that the solar ice maker is in the optimal operation state.

### 4.2. Effect of Condensation Temperature on System Performance

**Figure 4:**
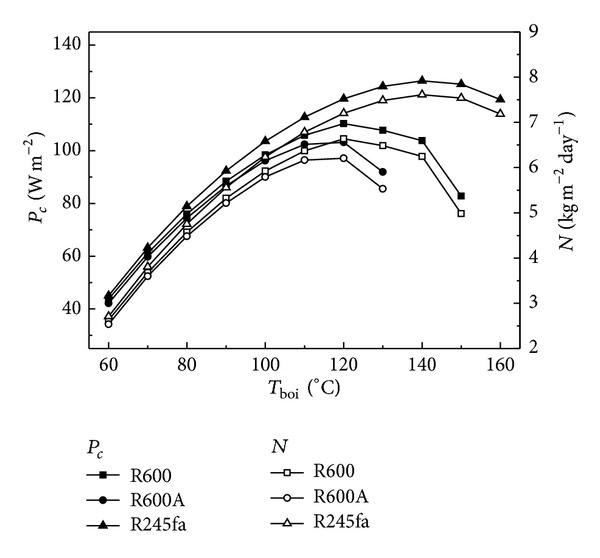
Effects of *T*_boi_ on *P*_*c*_ and *N*.

**Figure 5:**
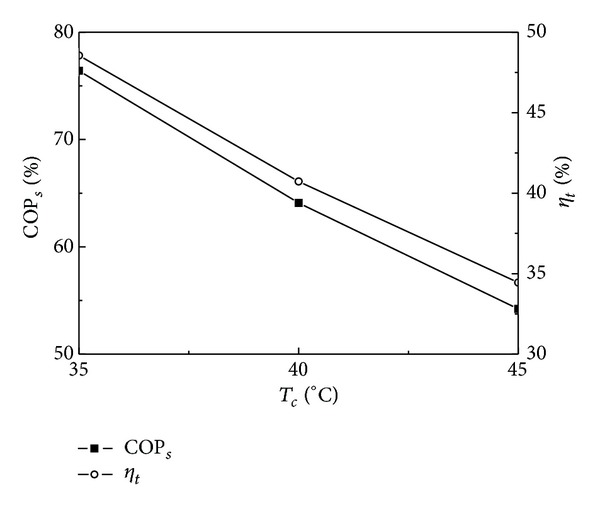
Effects of *T*_*c*_ on COP_*s*_ and *η*_*t*_.

The condensation temperature varies with ambient and the effects of condensation temperature on COP_*s*_, *η*_*t*_, *P*_*c*_, and *N* are shown in Figures 4 and 5. In Figures 4 and 5, the working fluid is R245fa and the generation temperature is 140°C. It is obvious from Figures 4 and 5 that COP_*s*_, *η*_*t*_, *P*_*c*_, and *N* decrease with the increase of *T*_*c*_. COP_*s*_ is 76.40%, 64.09%, and 54.21%, *η*_*t*_ is 48.55%, 40.72%, and 34.45%, *P*_*c*_ is 150.49, 126.44, and 107.13 W m^−2^, and *N* is 9.06, 7.61, and 6.45 kg m^−2^ day^−1^ when *T*_*c*_ is 35, 40, and 45°C, respectively. *P*_*c*_ at condensation temperature of 35°C is 1.19 times larger than that at 40°C and is 1.40 times larger than that at 45°C. Observing the profiles from Figures 4 and 5 it can be noticed that the condensation temperature has an important influence on COP_*s*_, *η*_*t*_, *P*_*c*_, and *N*.

The condensation temperature for air cooled condenser is about 45°C and for water cooled condenser is in the range of 35–40°C; using seawater especially as cooling medium the condensation temperature is about 35°C. Generally speaking, the condensation temperature for air cooled condenser is higher than that for water cooled condenser, resulting in the lower *N* for air cooled condenser. However, the power consumption and system investment for air cooled condenser are also lower than those for water cooled condenser which needs to be equipped with cooling water pump or cooling tower. Thus the system performance and payback period should be comprehensively considered so as to decide which type of condenser should be used during practical design.

### 4.3. Effect of Working Fluid Types on System Performance

**Figure 6:**
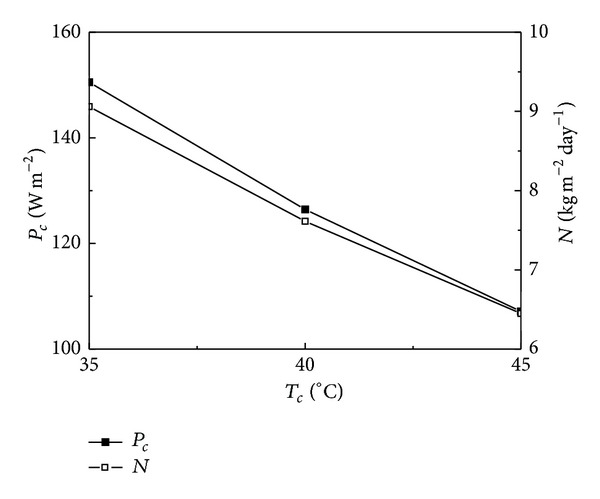
Effects of *T*_*c*_ on *P*_*c*_ and *N*.

**Figure 7:**
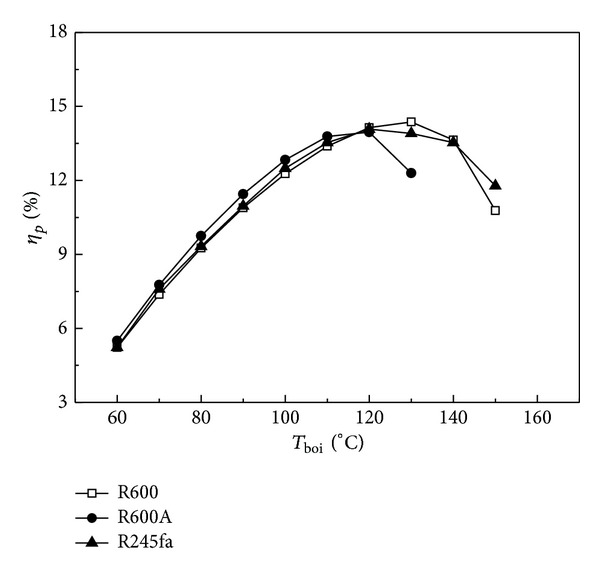
Effect of working fluid types on *η*_*p*_.

The system performance varies with different working fluids due to having different physical properties. *η*_*p*_ and COP_*c*_ are generally different when using different working fluids, leading to different COP_*s*_, as shown in Figure 6. The condensation temperature and evaporation temperature are 40 and −5°C in Figure 6, and the COP_*c*_ is, respectively, 3.63, 3.30, and 3.87 for working fluids of R600, R600A, and R245fa. As evident in Figure 6 *T*_boi_ has an important influence on *η*_*p*_, and *η*_*p*_ increases with *T*_boi_ when *T*_boi_ < 120°C. When *T*_boi_ < 110°C, *η*_*p*_ for working fluid R600A is superior to those for two other working fluids; however, R245fa has the maximum *η*_*p*_ when *T*_boi_ > 110°C. Observing the profiles from Figure 6, *η*_*p*_ achieves the maximum values at *T*_boi_ = 130, 120, and 150°C for working fluids of R600, R600A, and R245fa, respectively; that is to say, the ice making system employing working fluids of R600, R600A, and R245fa will not achieve the optimal performance when *T*_boi_ is higher than the temperature corresponding to the maximum *η*_*p*_.

To sum up, using organic Rankine cycle-vapor compression cycle for ice making driven by solar energy is feasible and the keys are to develop the expander and compressor with high efficiency, especially the compressor with high pressure ratio.

## 5. Conclusions

Solar powered ORC/VCC for ice making is researched and the effects of working fluid types, generation temperature, and condensation temperature on the system performance are analyzed by the development of a thermodynamic model, the following conclusions can be drawn.

1. The cooling power per square meter collector and ice production per square meter collector per day are, respectively, 126.44 W and 7.61 kg at the generation temperature of 140°C for working fluid of R245fa, which demonstrates the feasibility of solar powered organic Rankine-vapor compression ice maker.
2. COP_*s*_, *η*_*t*_, *P*_*c*_, and *N* depend largely on *T*_boi_ and they increase firstly and then decrease with increasing *T*_boi_, indicating that there is always an optimal *T*_boi_ at which *P*_*c*_ and *N* can achieve the maximum values, while *T*_boi_ can be controlled by adjusting the mass flow rate of working fluid for ORC.
3. The condensation temperature has an important influence on COP_*s*_, *η*_*t*_, *P*_*c*_, and *N*. *P*_*c*_ at condensation temperature of 35°C is 1.19 times larger than that at 40°C and is 1.40 times larger than that at 45°C, indicating that the ice making system equipped with air cooled condenser or water cooled condenser has different performances.
4. The system performance varies with different working fluids due to having different physical properties. For every working fluid there is an optimal *T*_boi_ at which *η*_*p*_ achieves the maximum values and the ice making system will not achieve the optimal performance when *T*_boi_ is higher than the optimal *T*_boi_.

## Figures and Tables

**Table 1:** Parameter values.

Parameter	*η*_exp⁡_	*η*_pump,*w*_	*η*_com_	*h*_ice_ (kJ kg^−1^)
Value	0.85	0.9	0.8	418.6

## Nomenclature

COP_*c*_: Coefficient of performance for VCC
COP_*s*_: Coefficient of performance for ORC/VCC
*G*_*B*_: Direct radiation intensity [Wm^−2^]
*h*_1_: Enthalpy at expander inlet [kJ kg^−1^]
*h*_2*s*_: Enthalpy at expander outlet based on isentropic process [kJ kg^−1^]
*h*_3_: Enthalpy at working fluid pump inlet [kJ kg^−1^]
*h*_4_: Enthalpy at working fluid pump outlet [kJ kg^−1^]
*h*_4*s*_: Enthalpy at working fluid pump outlet based on isentropic process [kJ kg^−1^]
*h*_5_: Enthalpy at evaporator outlet [kJ kg^−1^]
*h*_6*s*_: Enthalpy at compressor outlet based on isentropic process [kJ kg^−1^]
*h*_8_: Enthalpy at evaporator inlet [kJ kg^−1^]
*h*_ice_: Phase change latent heat of changing water into ice [kJ kg^−1^]
*m*_*c*_: Mass flow rate for VCC [kg s^−1^]
*m*_*p*_: Mass flow rate for ORC [kg s^−1^]
*n*: Collector area [m^2^]
*N*: Ice production per square meter collector per day [kgm^−2^ day^−1^]
*P*_*c*_: Cooling power per square meter collector [W m^−2^]
*Q*_boi_: Generator heat input [kW]
*Q*_eva_: Cooling power [kW]
*T*_boi_: Generation temperature in the generator [°C]
*T*_*c*_: Condensation temperature [°C]
*W*_com_: Compressor work input [kW]
*W*_exp⁡_: Expander work output [kW]
*W*_pump_: Working fluid pump power consumption [kW]
*W*_net_: Net work output for ORC [kW]
*η*_com_: Compressor isentropic efficiency
*η*_exp⁡_: Expander isentropic efficiency
*η*_*p*_: Organic Rankine cycle efficiency
*η*_pump_: Working fluid pump isentropic efficiency
*η*_solar_: Thermal efficiency of solar collector
*η*_*t*_: Overall efficiency of solar ice maker
Δ*T*: Temperature difference of inlet and outlet of collector [°C].
